# The Story of the Insane from Year to Year

**Published:** 1901-10-26

**Authors:** 


### THE STORY OF THE INSANE FROM YEAR TO YEAR.

(Thirteenth Series.)

### COTON HILL HOSPITAL FOR THE INSANE, STAFFORD.

One hundred and thirty patients were resident in this asylum on January 1st, 1899, and on December 31st the number had fallen to 128. There were 21 first admissions, 12 readmissions, 11 recoveries, 13 discharged relieved, and 5 deaths. On the men's side the recoveries stand at 18·8 per cent. of the admissions in that department, and on the women's side they stand at 47 per cent. This discrepancy shows how little the statistics of one year, or of one asylum, can tell us about the movements of the insane from which any reliable inferences can be drawn. The percentage of deaths on the average number resident was only 3·9. Of the year's admissions 10 are supposed to have owed their insanity to one or other of the various moral causes usually named in asylum statistics: drink accounts for 5, and hereditary influences, generally the most prominent figure, is absent altogether on this occasion. This hospital professes "to provide accommodation and comforts suitable to the former social and present mental condition of insane or nervous invalids of the middle and upper classes at moderate rates of payment," and that it fairly well justifies this profession may be gathered from the fact that last year fully 28 per cent. of the patients paid sums not exceeding one guinea a week, and that 68 per cent. were maintained for sums less than the average cost of their maintenance. Dr. Hewson remarks that these statistics do not warrant the committee in admitting patients at very low rates of payments and assuredly this is evident unless the subscriptions could be greatly increased, or preferably that some benevolent and rich individuals would add 50 beds to the institution. The average weekly cost of maintenance was £1 13s. per head, but with 200 patients in the asylum the rate would go down and hence charitable work could be increased. Does the rich county of Stafford not include 10 people able and willing to subscribe a total of £10,000 and carry this work out? We notice that the annual subscriptions amount only to £21. This small sum shows how little interest is taken in the middle-class insane by the middle-classes themselves. The report is well printed and illustrated, being more like a carefully prepared American institutional report than an English one.

### THE THREE COUNTIES ASYLUM.

On January 1st, 1899, this asylum contained 1,105 patients, and by December 31st the number had fallen to 1,035, but this drop in the numbers is not owing to any dearth of the insane in the Three Counties, but to the transfer of 90 patients to the new asylum at St. Albans. There were 209 first admissions, 42 readmissions, 104 recoveries, 10 discharged relieved, and 127 deaths. The average number resident during the year was 1,039; and the total number of persons under care was 1,385. Calculated on the admissions the recoveries stand at 37 per cent., and calculated on the average number resident, the deaths stand at 12·22 per cent., a rather high death rate. Of the deaths 35 were caused by cerebral and spinal diseases, 14 by consumption, 7 by pneumonia, 1 by bronchitis, 4 by disease of lungs (particular disease not stated), and 3 by enteritis. Of the admissions, 12 owed their insanity to domestic trouble, 9 to adverse circumstances, 8 to religion, 34 to drink, and 73 to hereditary influence. After 25 years' service Dr. Swain sent in his resignation and obtained a retiring allowance, on which we offer him our congratulations and also our wishes for improved health, illness being the cause of his retiring. Dr. de Lisle, the senior assistant medical officer, reigns in his place. The assistant matron, Miss Braines, also resigned after 25 years service, and was awarded a superannuation allowance. The committee wisely decided to abolish the office of assistant matron and appointed a head nurse instead. The salary given to this officer is not high, as she has 500 patients to look after, and the pension given to the late assistant matron was so low, that if the head nurse is to be treated on the same scale it cannot be said that she has much to look forward to in her old age. The cost per head per week was 9s. 1¾d.

